# Impact of asylum interviews on the mental health of traumatized asylum seekers

**DOI:** 10.3402/ejpt.v6.26286

**Published:** 2015-09-01

**Authors:** Katrin Schock, Rita Rosner, Christine Knaevelsrud

**Affiliations:** 1 Center for Torture Victims, Berlin, Germany; 2 Department of Clinical Psychology and Psychotherapy, Freie Universität Berlin, Berlin, Germany; 3 Department of Clinical and Biological Psychology, Catholic University Eichstätt-Ingolstadt, Eichstätt, Germany

**Keywords:** Asylum seekers, asylum interview, trial, trauma, posttraumatic stress disorder

## Abstract

**Background:**

Asylum interviews within the asylum procedure are associated with psychological stress for traumatized asylum seekers. This study investigates the impact of asylum interviews on the mental health in a sample of 40 traumatized asylum seekers. The comparison group consisted of refugees (*N=*10) that had not been invited to an asylum interview. Additionally, the moderating effects of trial-related variables such as perceived justice of the trial, stress of giving testimony, and stress of waiting for the asylum interview were examined.

**Method:**

Participants were assessed on average 10 days before (t1) and 16 days after (t2) the asylum interview. Chi-square tests for dichotomous and categorical variables were used to compare the descriptive statistics of the two groups. To investigate symptom changes from t1 to t2, paired *t*-tests were calculated. The magnitude of effects was measured by Cohen's effect size *d* within groups. Hierarchical regression analyses were conducted for demographic and trial variables predicting posttraumatic intrusions, avoidance, and hyperarousal.

**Results:**

Data showed a significant increase in posttraumatic intrusions and a significant decrease in posttraumatic avoidance and hyperarousal symptoms from t1 to t2. No significant symptom changes in the posttraumatic stress disorder subscales were found in the comparison group. The results of hierarchical regression analyses revealed perceived justice of the interview to predict the increase of intrusions and the number of experienced traumata and testimony stress to predict posttraumatic avoidance.

**Conclusions:**

The present findings underline the stressful impact of asylum interviews on traumatized refugees. They indicate that the asylum interview might decrease posttraumatic avoidance and trigger posttraumatic intrusions, thus highlight the importance of ensuring that the already vulnerable group of traumatized refugees needs to be treated with empathy during their asylum interview.

In Germany, 202,384 applications for asylum were made in 2014 (Federal Office for Migration and Refugees [BAMF], 2014), many of them by people claiming to have been forced to leave their home countries because of fear of persecution and human rights abuses. Many refugees and asylum seekers are traumatized by war, political/ethnical oppression, or torture. In response to the increasing numbers of immigrants, typical hosting areas, such as Europe, North America, and Australia, have tightened their immigration laws and asylum procedures in recent years. Only a small number of those seeking asylum are recognized as refugees (BAMF, 2014). Those rejected face repatriation to their countries of origin, where they believe themselves to be at risk of harm or persecution.

Refugees and asylum seekers are highly vulnerable to psychological disorders (Fazel, Wheeler, & Danesh, 2005; Johnson & Thompson, 2008; Terheggen, Stroebe, & Kleber, 2001). Posttraumatic stress disorder (PTSD) is one of the most frequent disorders in the aftermath of a traumatic event. PTSD is characterized by intrusions, including remembering the traumatic situation recurrently and involuntarily; avoidance of trauma-associated thoughts, situations, and actions; and hyperarousal, which is reflected in psychovegetative symptoms such as sleeping disorders, difficulty concentrating, and elevated heart rate (DSM-IV; American Psychiatric Association [APA], 1994). In the revised version of the DSM-5, the authors propose four distinct diagnostic clusters instead of three for diagnosing PTSD. They are described as re-experiencing, avoidance, negative cognitions and mood, and arousal (DSM-5; APA, 2013).

Empirical studies on the lifetime prevalence of PTSD among asylum seekers and refugees have reported rates up to 65% (Hinton et al., 2006; Keller et al., 2003). Refugee populations experience traumatic events, such as torture and war, with disproportionate frequency prior to their displacement (Hargreaves, 2002; Hollifield et al., 2002). PTSD prevalence rates among refugees who have experienced torture range from 18 to 85% (Johnson & Thompson, 2007).

In addition to their premigration trauma, individuals with temporary resident status experience substantial postmigration stress (Jamil, Nassar-McMillan, & Lambert, 2007; Silove et al., 2007; Teodorescu, Heir, Hauff, Wentzel-Larsen, & Lien, 2012), including insecurity regarding their legal status (Nickerson, Steel, Bryant, Brooks, & Silove, 2011; Ryan, Benson, & Dooley, 2008; Silove et al., 2007) and lasting fear of repatriation and persecution (Herlihy, Scragg, & Turner, 2002; Nickerson et al., 2011; Steel, Frommer, & Silove, 2004). These postmigration factors can also have adverse effects on the mental health of asylum seekers (Carswell, Blackburn, & Barker, 2011; Laban, Gernaat, Komproe, & De Jong, 2007).

As traumatized refugees and survivors of torture are a highly vulnerable group, many need time to process past traumatic events and to establish a sufficient level of trust and confidence before they are able to reveal the potentially painful and shaming details of their experiences (Bogner, Herlihy, & Brewin, 2007). Instead, after their arrival in Germany, the immigration authorities interview refugees applying for asylum. In this asylum interview, the refugees have a “duty to cooperate”: they are obliged to disclose all facts related to their asylum application, including the history of their persecution and their reasons for fleeing their home country. This asylum interview has been identified as a postmigration stressor (Laban, Gernaat, Komproe, Van der Twel, & De Jong, 2004). Previous studies have shown that most asylum seekers experience the immigration process—including the asylum interview—as being stressful and provoking anxiety. One reason may be the fear of consequences such as deportation (Bogner et al., 2007; Nickerson et al., 2011). Apart from potential adverse outcomes, the experience of the hearing itself may also cause significant distress. Being confronted with traumatic memories may activate symptoms of PTSD during the hearing (Bogner et al., 2007). Moreover, having to report the traumatic event in detail during court hearings may aggravate PTSD symptoms in victims of violence with PTSD (Koss, 2000).

Empirical findings on the impact of court hearings on psychological outcomes are inconclusive. There are studies on the impact of the refugee determination process on the mental health of asylum seekers (Hocking, Kennedy, & Sundram, 2015; Mueller, Schmid, Staehli, & Maier, 2011; Silove et al., 2007). But to our knowledge no previous empirical study has examined explicitly the impact of asylum interviews on the mental health of asylum seekers and refugees. In order to provide an overview of the consequences of testifying in court we review existing studies examining people who were victims of different crimes below.

In a study of women with a history of sexual abuse (*N=*288), Epstein, Saunders, and Kilpatrick (1997) reported adverse effects of testifying in court: Of those who testified in court, 64% developed PTSD, whereas only 24% of those who did not testify in court met the criteria for PTSD symptoms. However, including the severity of the incident as a predictor in a multiple regression analysis of PTSD status shows that having testified was no longer a statistically significant predictor. This result is in line with the findings of a study of victims of robbery (*N=*74), in which Hammer (1989) found that testifying in a trial hearing is not associated with a significant increase in PTSD symptoms. Likewise, in a study of rape victims (*N=*569), Frazier and Haney (1996) found no significant increase in symptoms after testifying, and neither attitudes nor case outcomes were associated with victims’ post-rape recovery. Orth and Maercker (2004) found no significant increase in PTSD symptoms in a study of victims of sexual violence who testified in court. In contrast, legal secondary victimization (e.g., in cases where the victim was informed by the police that the case was not serious enough to pursue) was positively associated with posttraumatic stress reactions in a study of rape victims (*N=*102; Campbell et al., 1999).

In terms of general psychological distress, crime victims have been found to experience elevated levels of distress subsequent to their testimony in court. Orth and Maercker (2004) demonstrated that renewed feelings of powerlessness and perceived unfair treatment moderated the level of distress experienced. McFarlane (1996) examined the psychological effects of court hearings on victims of a natural disaster (*N=*32) and reported that 57% of the victims experienced the civil litigation process as traumatizing or distressing. The delay between reporting a crime to the police and the start of the trial has been identified as a significant predictor of victims’ evaluation of the litigation process as traumatizing (Gutheil, Bursztajn, Brodsky, & Strasburger, 2000).

Empirical findings on the impact of court hearings on psychological outcomes are thus inconclusive. It is important to differentiate between the meaning of the outcome of an asylum claim and the one of crime victims because it is possible that asylum seekers will be repatriated (or detained) if their claim is rejected. This makes it problematic to directly extend the results of studies on crime victims to the literature on refugees and asylum seekers and illustrates the importance of conducting this study.

## Objective

In our clinical experience, asylum seekers often report a worsening of their clinical symptoms after having gone through the asylum interview. We conducted this study to verify this subjective impression and to investigate influencing factors during the asylum interview. The first aim of our study was therefore to examine the impact of the asylum interview on the mental health of traumatized refugees and survivors of torture with PTSD, depression, and anxiety symptoms. The second aim was to investigate the moderating influence on symptom severity of trial-related variables such as the perceived justice of the hearing, the stress of giving testimony (testimony stress), and the stress of waiting for the asylum interview (delay stress).

We hypothesized first that the more asylum seekers experience the asylum interview as unjust, the higher will be the overall increase in PTSD symptoms, anxiety, and depressive symptoms. Second, we predicted that the more testimony stress is caused by giving testimony about the traumatic experience, the higher the increase in PTSD symptoms, especially intrusive symptoms, and anxiety and depression.

## Methods

### Sample groups

Participants in the present study were traumatized asylum seekers who fled to Germany as refugees, had applied to the BAMF for asylum, and whose asylum interview at the BAMF took place between the first and second occasions of measurement (asylum group, AG). They were recruited through specialized treatment centers (*N=*37) and lawyers (*N=*10). In a first contact (face-to-face or by phone, facilitated by a trained interpreter), 47 interested participants were informed about the background and procedures of the study. They were informed that their participation would not affect the outcome of their asylum interview in any way. Furthermore, they were assured that their participation was voluntary and that all data would be treated confidentially. Participants also received this information in written form. Four of the asylum seekers, who had been recruited through lawyers, declined to participate in the study. Three of them (all men) stated that they remained doubtful that the results of the study would have no negative effect on the outcome of their asylum claim. One woman had no wish to talk about her experiences and feelings with an interviewer she did not know. Asylum seekers recruited through treatment centers agreed to participate. Thus, 43 asylum seekers gave their approval to take part in the study. All participants provided signed informed consent before the first assessment point. The assessment interviews were conducted in the Treatment Center for Torture Victims in Berlin by trained psychologists and facilitated by trained and sworn interpreters, who had a wealth of experience in working with refugees. The comparison group consisted of refugees (*N=*10) who were waiting to begin therapy in the treatment center, but had not yet been invited to a hearing at the BAMF, and whose asylum interview did not take place between the two assessment points. The inclusion criteria for the comparison group were a PTSD symptom level at assessment point one of a PDS sum-score of >30 (severe symptom level; Posttraumatic Diagnostic Scale, Foa, 1995; Foa, Cashman, Jaycox, & Perry, 1997), a minimum of five experienced traumatic events, a minimum age of 18 years, and a level of education of at least 5 years. The small size of this sample group is explained by the fact that very few asylum seekers who seek assistance in treatment centers for refugees and torture survivors have not received an invitation for the hearing at that point in time. On average, the asylum seekers who passed through the asylum interview (AG) had entered Germany 7 months before the interview (SD*=*6.3 months). The asylum seekers of the comparison group (CG), in contrast, had entered Germany 5 months (SD*=*4.8 months) before the first assessment point. The interviews were conducted, on average, 10 days (SD*=*9 days) before the asylum interview (t1) and 16 days (SD*=*13 days) after the interview (t2). The Ethics Committee of the German Psychological Association approved the study.

All the participants recruited from treatment centers took part in the second assessment. Of those recruited through lawyers, three did not take part in the second assessment. The asylum interviews of two of those participants were postponed to a later date and the third was no longer reachable. In all, 40 asylum seekers were included in the statistical analysis.

### Measures

Demographic data including age, sex, level of education, marital status, and time since arrival in Germany were collected from each participant (Table 1). Mental health variables were assessed by standardized questionnaires. The measures on psychopathology were all translated from the German and then retranslated in advance of the study in order to ensure the accuracy and content validity of the scale items.

**Table 1:** Demographic data for the asylum group (AG; *n=*40) and the comparison group (CG; *n=*10)

	AG		CG		Group comparison
	
	*N*	%	*N*	%	
Sex (female)	17	43	3	30	*χ* ^2^=0.52, df=1, *p*=0.47
Country of origin					*χ* ^2^=14.01, df=10, *p*=0.17
Iran	12	30	3	30	
Balkans	7	18	–	–	
Turkey	6	15	3	30	
Chechnya	6	15	1	10	
Others	9	22	3	30	
Marital status					*χ* ^2^=1.29, df=1, *p*=0.47
Single	20	50	7	70	
Married	20	50	3	30	
	Mean	SD	Mean	SD	
Age in years	32.57	10.16	30.0	11.11	*t=*0.53, *p*=0.60
Level of education	8.4	2.8	7.9	2.3	*t=*0.70, *p*=0.45
No. of traumatic events	17	3.6	16	2.4	*U=*0.72, *ns*
No. of directly experienced traumatic events	10	2.9	9	2.3	*U=*0.14, *ns*

#### Traumatic events

Participants identified the trauma they had experienced and/or witnessed on a traumatic event list consisting of 23 items. The items were derived from the PDS Part I (Foa, 1995) and complemented by items from the Harvard Trauma Questionnaire event list (Mollica et al., 1992), and additional traumatic events relevant for traumatized refugees and torture survivors like “brainwashing.”

#### Posttraumatic stress disorder

When the present study was conducted, the diagnostic criteria for PTSD found in DSM-IV PTSD still applied. Hence, we rely here on the DSM-IV PTSD criteria rather than those in DSM-5. PDS (Foa, 1995; Foa, et al., 1997) was used to assess symptoms of posttraumatic stress. This self-report questionnaire consists of a traumatic event scale and a symptom scale. The symptom scale comprises the subscales “intrusions,” “avoidance,” and “hyperarousal.” It is closely modeled on the DSM-IV criteria for PTSD and can be administered repeatedly to help monitor symptom change (APA, 1994). The scale has very good internal consistency (*α*=0.92), test–retest reliability (*r=*0.74) (Briere, 1997), and has been validated across several populations. Participants were asked to indicate the frequency of each symptom over the past 4 weeks on a four-point scale ranging from 0=*never* to 3=*daily*. The PDS functions well compared to clinician ratings; it is seen as a useful self-report and screening device for measuring PTSD and its component symptoms (Keane & Kaloupek, 2002).

#### Anxiety and depression

Symptoms of anxiety and depression were assessed with the Hopkins Symptom Checklist (HSCL-25; Derogatis, Lipman, Rickels, Uhlenhuth, & Covi, 1974; Mollica, Wyshak, De Marneffe, Khoun, & Lavelle, 1987). The scale was adapted for use in refugee populations and has very good validity and reliability (Mollica et al., 1987). The internal consistency (Cronbach's *α*) is 0.84 for the anxiety scale and 0.91 for the depressive scale. Respondents were asked to indicate the extent to which they had been bothered by each symptom in the previous week on a four-point scale from 1=*not at all* to 4=*extremely*.

#### Trial-related variables

To investigate how other factors—such as the perceived justice of the hearing, the stress of giving testimony, and the stress of waiting for the hearing—might influence changes in symptom severity we modified and adapted the Trial Variables Questionnaire by Orth and Maercker (2004) to apply specifically to the asylum interview. This questionnaire, which was developed and used in a study on the impact of court hearings on traumatized victims (Orth & Maercker, 2004), consists of eight scales measuring the subjective experience of trials. We used three of the scales: “perceived justice of the hearing” was assessed using a scale with six items, such as *The interviewer treated me courteously and respectfully*. “Psychological stress” caused by giving testimony, which included only one item in the original version of Orth and Maercker (2004) (*Giving testimony was stressful to me*), was supplemented by four items to elicit more precise information about what was stressful in giving testimony. An item example is: *I had to talk about experiences I was ashamed for*. The scale of psychological stress caused by the delay until the beginning of the asylum interview was assessed with a single item: *It was stressful to me to wait for the beginning of the asylum interview*. Responses were given on a six-point Likert scale ranging from 0=*strongly disagree* to 5=*strongly agree*.

### Data analysis

Statistical analyses were performed using the Statistical Package for the Social Sciences, version 22.0 (SPSS 22.0) for MAC. Chi-square tests for dichotomous and categorical variables, either the *t*-test for independent variables, or the Mann–Whitney *U*-test were used to compare the descriptive statistics of the two groups. To investigate symptom changes from t1 to t2, paired *t*-tests were calculated. The magnitude of effects was measured by Cohen's effect size *d* within groups; this is calculated as the difference between the means at t1 and t2, divided by a standard deviation for the data. Cohen defined effect sizes as small (*d=*0.20), medium (*d=*0.50), and large (*d=*0.80). Data are reported as means and standard deviations.

We conducted a hierarchical regression analysis for demographic and trial variables predicting posttraumatic intrusions, avoidance, and hyperarousal. Data were tested for normal distribution using the Kolmogorov–Smirnov test.

## Results

Table 1 summarizes the demographic characteristics of the two groups with (AG) or without (CG) an asylum interview. There are no essential group differences between the asylum group and the comparison group participants.

Whereas half of the participants in the AG were married (*n*=20, 50%), about two-thirds of those in the CG were single (*n*=7, 70%). Most participants in the AG group came from Iran (*n*=12, 30%; CG: *n*=3, 30%), followed by the Balkan region (*n*=7, 17.5%). The language groups the participants were drawn from were Farsi (Persian), Russian, Turkish/Kurmanci (Kurdish), and English.

Participants reported on average 17 traumatic events (range 7–22), 10 of which were direct personal experiences of trauma (range 2–16), as opposed to traumas witnessed or learned about. The most frequently reported directly experienced traumatic events included high impact traumata such as torture (AG: 70%, CG: 90%) and imprisonment (AG: 60%, CG: 80%). As shown in Table 2, participants’ responses revealed high levels of psychological distress at t1. The average PDS score was *M=*35.16 (SD=5.79) in the AG and *M* = 34.6 (SD*=*9.1) in the CG, indicating considerable symptom severity. Similarly, both samples reported high anxiety and depression severity. Average scores on the HSCL-25 anxiety subscale were *M*=2.81 (SD=0.47) in the AG and *M*=2.86 (SD=0.51) in the CG; those on the HSCL-25 depression subscale were *M*=3.00 (SD=0.55) in the AG and *M*=2.96 (SD=0.56) in the CG. A group comparison on symptom severity at the first assessment (t1) did not differ significantly on any of the psychopathology subscales.

**Table 2:** Means (standard deviations) of the outcome variables at assessment point one (t1, before the asylum interview) and two (t2, after the asylum interview) by group

		t1 (before the interview)	t2 (after the interview)	ES t1 to t2
Posttraumatic Stress Diagnostic Scale				
Intrusions	AG	10.72 (2.32)^a^	13.52 (1.78)^b^	1.38
	CG	11.50 (3.43)^a^	10.9 (2.37)^a^	0.20
Avoidance	AG	13.46 (3.76)^a^	12.10 (3.18)^b^	0.39
	CG	12.60 (4.27)^a^	14.30 (4.39)^a^	0.39
Hyperarousal	AG	10.97 (2.81)^a^	9.35 (2.51)^b^	0.61
	CG	10.50 (3.43)^a^	10.60 (3.43)^a^	0.02
PTSD-sum	AG	35.16 (5.79)^a^	34.97 (5.59)^a^	0.03
	CG	34.60 (9.1)^a^	35.8 (8.97)^a^	0.13
Hopkins Symptom Checklist-25—Anxiety subscale	AG	2.81 (0.47)^a^	2.69 (0.56)^a^	0.23
	CG	2.86 (0.51)^a^	2.60 (0.43)^b^	0.55
Hopkins Symptom Checklist-25—Depression subscale	AG	3.00 (0.55)^a^	2.60 (0.77)^b^	0.15
	CG	2.96 (0.56)^a^	2.71 (0.43)^a^	0.59

a,b Means within a row that share a superscript do not differ at *p<*0.05.

AG=asylum interview group; CG=comparison group; ES=effect size Cohen's *d*.

Table 2 presents means and standard deviations of all symptoms at the two assessment points and Cohen's *d* effect sizes for symptom changes from t1 to t2 within each group. The data revealed significant t1 to t2 changes in the PTSD subscales in the asylum group (AG): the intrusion subscale showed a significant increase after the asylum interview and a large effect size (*t*(39)=6.87, *p=*0.000; *d=*1.38), whereas avoidance subscales (*t*(39)=2.03, *p*=0.04; *d=*0.39) and hyperarousal (*t*(39)=3.11, *p=*0.003; *d=*0.61) indicate a significant symptom decrease with small and medium effect sizes. In contrast, there were no significant symptom changes in the PTSD subscales in the comparison group (CG). Furthermore, the AG showed a significant decrease in depressive symptoms following the asylum interview (*t*(39)=2.89, *p=*0.006; *d=*0.15), whereas no significant depression symptom changes were found in the CG. Anxiety symptoms in the CG decreased significantly (*t*(9)=6.87, *p=*0.000; *d=*0.55), but not in the AG (*t*(39)=2.29, *p=*0.04; *d=*0.55).

We conducted a hierarchical regression analysis predicting posttraumatic intrusions, avoidance, and hyperarousal after testing the requirements for it. Table 3 shows the summary of this analysis. Time 1 psychological symptoms were controlled for. To control the demographic variables, age, education, and number of experienced traumata were entered simultaneously into the regression equation in step 1. In step 2, the variables perceived justice of the hearing, stress of giving testimony (testimony stress), and stress of waiting for the beginning of the hearing (delay stress) were added to test the variance explained by these trial variables. Perceived justice of the hearing qualifies for the regression equation with a regression coefficient of *β*=−0.33 (*p=*0.02) in step 2 (*R* ^2^ – change*=*0.29, *p=*0.01). The direction of all regression coefficients was as predicted. The results of hierarchical regression analysis predicting posttraumatic avoidance reveal that the number of experienced traumata (*β*=0.44, *p=*0.01) and testimony stress (*β*=0.43, *p=*0.02) significantly increase the variance explained in step 2 (*R* ^2^ – change=0.32, *p=*0.02). Concerning the regression analysis predicting posttraumatic hyperarousal no significance was found in either step 1 (*R* ^2^ – change=0.08, *p=*0.39) and step 2 (*R* ^2^ – change=0.17, *p=*0.14).

**Table 3:** Predictors of increase in posttraumatic intrusions after the asylum interview

	*B*	*SE B*	*β*
Step 1			
Constant	11.07	0.88	
Age	0.03	0.03	0.20
Education in years	−0.12	0.12	−0.19
No. of traumatic events	0.12	0.11	0.19
Step 2			
Constant	9.87	1.01	
Age	0.04	0.03	0.24
Education in years	−0.18	0.11	−0.29
No. of traumatic events	0.06	0.10	0.08
Testimony stress	0.05	0.07	0.11
Delay stress	0.34	0.18	0.29
Perceived Justice	−0.19	0.10	−0.33*

*R* ^2^ *=*0.11 for Step 1; Δ*R* ^2^=0.29 for Step 2 (*p<*0.05).

* *p<*0.05.

## Discussion

To our knowledge this is the first study to examine how the substantive interview within the asylum process influences the mental health of traumatized asylum seekers. It is known that many asylum seekers suffer severe mental health problems (Fazel et al., 2005). Our study examined asylum seekers who had experienced high impact traumata in the past and thus suffered from severe mental health problems. We assessed PTSD subscales, anxiety, and depressive symptoms at the baseline assessment point before the asylum interview and again after the interview. A main focus of interest was on how trial variables such as perceived justice during the asylum interview, experienced stress in giving testimony reporting traumatic experiences, and experienced stress of waiting for the beginning of the asylum interview, influence the impact of the interview on the psychopathology. We found a significant increase in the posttraumatic intrusions and significant decrease in posttraumatic avoidance and hyperarousal symptoms after the asylum interview. Previous studies on the specific nature of intrusions provide a possible explanation, because they suggested that intrusive memories are stimuli that acquire the status of warning signals through temporal association with the trauma—that is, stimuli that, if encountered again, would indicate impending danger (Ehlers et al., 2002). Although criterion A1 of the DSM-IV does not define an asylum interview as a traumatic event (APA, 1994), the asylum seekers in our study were severely traumatized by premigratory factors such as surviving war and torture. The situation may evidently remind them of life-threatening experiences in their home country—and thus may stimulate and reactivate the associated feelings of helplessness. The feeling of being ruthlessly exposed to a situation and helpless to change it (criterion A2, DSM-IV; APA, 1994) has been identified as a factor determining how traumatic a situation is perceived to be (Robinson & Larson, 2010). Trauma-related stimuli may thus lead to an increase in PTSD when they are associated with life-threatening situations.

The increase of posttraumatic intrusions may be related to the identified decrease of posttraumatic avoidance symptoms. Herlihy and Turner (2006) argued that avoidance initially serves as a survival strategy. According to these authors, many refugees state that they only manage to escape their situation and to cope with migration to a new country by deliberately avoiding thinking about what happened during the traumatizing event. This strategy is undermined during a court hearing, especially during the testimony phase (Pitman, Sparr, Saunders, & McFarlane, 1996). Thus, the avoidance of reminders of the traumatic events cannot be maintained as a coping mechanism during the asylum interview, which may be very distressing. From a therapeutic perspective, reducing avoidance is the pivotal mechanism of psychotherapy with PTSD patients (Varra & Follette, 2005). The crucial difference between the two contexts is that avoidance is forcibly penetrated during the asylum interview and that the individual does not experience the ability to cope with the memories of the traumatic event. This may reinforce stress or even be detrimental to the individual, also because of a possibly uncontrolled excess of intrusions. For example, high levels of dissociation during asylum interviews were reported by participants in Bogner's et al. (2007) study.

The significant decrease in posttraumatic hyperarousal symptoms in the AG might be due to a temporary feeling of relief after passing the asylum interview. A feeling of having overcome a crucial step towards safety in the country of exile, the so-called “honeymoon effect,” may also be assumed to contribute to the decrease in hyperarousal symptoms. In contrast, there were no significant PTSD symptom changes in the comparison group.

We found further that the perceived justice of the hearing predicts the increase of intrusions. Previous studies indicate that, besides a lack of social support, the lack of social acknowledgement of the traumatic experience predicts the development of PTSD (Brewin, Andrews, & Valentine, 2000). The experience that one's account is not believed and that one's suffering is invalidated may result in feelings of powerlessness, humiliation, helplessness, and fear, potentially leading to increased intrusions. Intrusions are associated with significant psychological distress (Simms, Gros, Watson, & O'Hara, 2008), and they can be triggered by asylum interviews.

We are aware that the present study has some weak points and is limited by several factors. Because no third assessment point was implemented, we cannot draw conclusions about the duration of the increase in intrusions after the asylum interview. Neither do we know whether or not the study participants received residence permits. Previous studies showed sharp reductions in PTSD symptoms following resolution, in the present case the granting of asylum. However, the stressful nature of a pending asylum claim exacerbates psychological distress in general, and thus increases PTSD scores. Because the participants did not yet know about their resident status when we assessed them the second time, we obviously cannot draw any conclusions about the relationship between resident status and PTSD symptoms. The results of previous studies on the impact of the asylum decision on PTSD symptoms are inconsistent. Some have found that a positive outcome of the hearing is accompanied by significantly reduced PTSD symptoms (Davis & Davis, 2006; Silove et al., 2007; Steel et al., 2011). On the other hand, in a longitudinal study, Ruf et al. (2007) showed that attaining permanent resident status was associated with a decrease in symptoms of depression but had no influence on PTSD. Because the decision for permanent residency can take months to come, we cannot speculate on whether a positive outcome may have been associated with an increase in intrusions over time.

Moreover, the comparison group was small, because very few asylum seekers who look for assistance in a center for the treatment of torture survivors and refugees have not yet received an invitation for the asylum interview by that time. This was the condition for inclusion in the comparison group. Unlike the asylum group, these participants did not receive therapeutic treatment during the study period, whereas almost all members of the asylum group (*n*=37, 92.5%) were in psychotherapy during this time. In other words, they were informed about the asylum interview process and supervised subsequent to it. Against this background, we cannot draw any conclusions on the natural course of symptoms during the asylum process.

## Conclusions

Our results have implications for both decision-makers and clinicians. The increased number of wars and armed conflicts in the world leads to continually increasing numbers of refugees and asylum seekers looking for secure residence in western countries. Many asylum seekers are highly traumatized and thus suffer from severe physical and mental health problems. Stress factors after the asylum interview might affect the recuperation process, the processing of the traumata and foster chronification of the symptoms. The asylum process with its asylum interview is seen as one of the postmigration stressors asylum seekers have to deal with. Our study results give an idea of the high vulnerability of traumatized asylum seekers and show that the asylum interview might decrease posttraumatic avoidance and trigger posttraumatic intrusions. This is even more remarkable as our study participants received support for the preparation of their asylum interviews, e.g., normally they have already talked about traumatic experiences before the asylum interview. Without preparation and psychological support there might be an even greater symptom increase. An elaborated and sensitive preparation for the asylum interview and postprocessing by clinicians after the interview or hearing are important in order to avoid permanent symptom increase or even chronification of the symptoms. The results of a study conducted by Gäbel, Ruf, Schauer, Odenwald, and Neuner (2005) showed that, even after psychological training, official judicial interviewers were unable to identify PTSD cases during asylum interviews.

It would make sense to provide the responsible decision-makers with special training to gain psychological knowledge and sufficient understanding of the effects of PTSD, thus heightening their awareness and helping them to better understand the specific needs of traumatized refugees in the context of a hearing. Not only could such awareness reduce the effects of distress on memory performance, it could also enhance the asylum seeker's perception of the justice of the asylum interview.
